# Quality and Cost of Diabetes Mellitus Care in Community Health Centers in the United States

**DOI:** 10.1371/journal.pone.0144075

**Published:** 2015-12-04

**Authors:** Patrick Richard, Peter Shin, Tishra Beeson, Laura S. Burke, Susan F. Wood, Sara Rosenbaum

**Affiliations:** 1 Department of Preventive Medicine and Biometrics, Uniformed Services University of the Health Sciences, Bethesda, Maryland, United States of America; 2 Department of Health Policy and Management, Milken Institute School of Public Health, The George Washington University, Washington, District of Columbia, United States of America; 3 Central Washington University, Ellensburg, Washington, United States of America; Medical University Vienna, AUSTRIA

## Abstract

**Objective:**

To examine variations in the quality and cost of care provided to patients with diabetes mellitus by Community Health Centers (CHCs) compared to other primary care settings.

**Research Design and Methods:**

We used data from the 2005–2008 Medical Expenditure Panel Survey (N = 2,108). We used two dependent variables: quality of care and ambulatory care expenditures. Our primary independent variable was whether the respondent received care in a Community Health Centers (CHCs) or not. We estimated logistic regression models to determine the probability of quality of care, and used generalized linear models with log link and gamma distribution to predict expenditures for CHC users compared to non-users of CHCs, conditional on patients with positive expenditures.

**Results:**

Results showed that variations of quality between CHC users and non-CHC users were not statistically significant. Patients with diabetes mellitus who used CHCs saved payers and individuals approximately $1,656 in ambulatory care costs compared to non-users of CHCs.

**Conclusions:**

These findings suggest an opportunity for policymakers to control costs for diabetes mellitus patients without having a negative impact on quality of care.

## Introduction

Studies have shown that community health centers (CHCs) provide care to patients at lower costs than other primary care settings, but researchers are still debating if CHCs achieve these cost savings by providing lower quality of care to patients compared to other primary care settings [1–4] While most studies have found that CHCs provide similar or better quality of care to patients, other studies have found that CHCs provide a lower quality of diabetes mellitus care or of primary care to patients compared to those receiving care in private offices [5–8]. Variations in sample size and measures of quality of care including subjective measures of patient satisfaction may explain the inconsistent findings [9–10]. Our study design and methodology attempt to address some of these shortcomings by using nationally representative data sets and more objective measures of quality of care. Hence, the research question for the current study is as follows: are there any significant differences in the quality and cost of care provided to patients with diabetes mellitus by Community Health Centers (CHCs) compared to other primary care settings?

The federal government has invested considerable resources in expanding CHCs for the last several years to provide primary care and preventive services to low-income individuals, including those with chronic conditions such as diabetes mellitus [11]. The Patient Protection and Affordable Care Act (ACA) provides an additional $11 billion in mandatory funding from 2011 to 2015 to double the capacity of health centers in the coming years [11]. Recent analyses by Sharac et al (2015) using 2014 data from the Uniform Data System (UDS) showed that CHCs represent the single largest comprehensive primary health care system serving medically underserved communities in the United States, in more than 9,000 urban and rural locations. CHCs provide comprehensive primary care to about 23 million patients who are either low-income, Medicaid beneficiaries or uninsured [11]. For instance, seventy percent of these patients have household incomes at or below 100 percent of the federal poverty level [11].

CHCs may reduce the demand for more expensive forms of care, such as outpatient specialty care, emergency care or inpatient hospitalizations for diabetes mellitus patients by providing access to quality primary care services for low-income patients, who are largely uninsured or on Medicaid. Several studies found that the usage of CHCs in underserved areas has been associated with lower hospitalization rates, fewer Emergency Department (ED) visits, and shorter inpatient stays [4]. Medicaid beneficiaries that use CHCs are less likely to have ED visits, be hospitalized or have recurring admissions for ambulatory care sensitive conditions [12]. Preventable hospitalizations were also found to be associated with the availability of ambulatory care clinics for vulnerable groups and elderly in medically underserved areas [4], where higher availability of affordable primary care translated into less preventable hospitalizations.

It is important for policymakers and researchers to understand whether or not CHCs achieve these cost savings at the expense of quality of care provided to patients with diabetes mellitus because diabetes mellitus is a costly chronic condition that disproportionately impacts racial/ethnic minorities and low-income populations that are served by CHCs [13–14]. Lower quality of care may result in poor management of diabetes mellitus, which may lead to serious co-morbidities including diabetic retinopathy, kidney failure, periodontal disease, and nervous system damage [13–14]. Further, findings from this study may inform the implementation of the ACA that seeks to contain healthcare expenditures while improving the quality of care for individuals with chronic conditions in the United States.

## Research Design and Methods

### Data Sources and Study Design

We used four years of recently available data (2005–2008) from the Medical Expenditure Panel Survey (MEPS). The MEPS is a nationally representative longitudinal survey that covers the United States civilian non-institutionalized population. We combined data from three different components of the MEPS including the Household Component (HC) file, the Office-Based Medical Providers Visits (OBMP) file, and the Outpatient Visits (OPV) file (see S1 Data). The HC file is the core component of the survey that collects demographic characteristics, health expenditures, health conditions, health status, medical services utilization, access to care, health insurance coverage, and income data for each person surveyed. The HC also contains the Diabetes mellitus Care Survey, a self-administered questionnaire (SAQ) to adult respondents 18 years or older who reported that they had been diagnosed with diabetes mellitus by a health care professional. The OBMP component collects data on dates of visits, diagnosis and procedure codes, charges and payments, and different types of office-based medical providers. Finally, the OPV component collects information on outpatient visits. The overlapping design of the MEPS allows repeated observations of the same people several times during the year. We combined data from the HC files with the pooled estimation linkage file of MEPS to restrict the analytic sample to unique individuals [14]. The analytic sample included a total of 2,108 individuals who were between 18 and 65 years old and reported that they had been diagnosed with diabetes mellitus by a healthcare professional.

### Dependent Variables

We used two dependent variables in this analysis: quality of care and ambulatory care expenditures.

#### Quality of Care

Consistent with the American Diabetes mellitus Association guidelines, we used three binary indicators to measure quality of care for patients with diabetes mellitus who reported receiving the following in the past year: two or more glycated hemoglobin (HbA1c) tests, foot examinations, and eye examinations [14]. These process measures are based on survey responses from the Diabetes mellitus Care Survey (DCS) supplement.

#### Ambulatory Care Expenditures

These expenditures are actual payments from all sources made by insurers and individuals as out-of-pocket costs for ambulatory care services, such as office-based services and non-emergency hospital outpatient care services. They do not include over-the-counter purchases. The MEPS subsequently validates dates of visits, diagnosis codes and procedure codes with providers and insurers.

### Independent Variables

Our primary independent variable was whether the respondent received care in a CHC or not. We coded CHC as 1 if the patient reported that they received more than 50% of their care from “community health centers” or “neighborhood/family health centers”. This definition of CHC does not include rural or free health clinics because the MEPS does not include information about these particular clinics. The CHC measure was constructed using the data from the event files to calculate the proportion of office-based visits and outpatient visits that respondents received in CHCs. Those individuals who reported that they received less than 50% of their care from CHCs were considered as receiving care from other primary care settings. Non-CHC users were those patients who received their primary care elsewhere (e.g. doctor’s office, group practice, medical clinic, managed care plan centers, company, school, hospital or other types of clinics) and were coded as zero.

### Control Variables

For the quality of care models, we controlled for a set of patient characteristics known to be associated with variation in quality of care including age, race/ethnicity, sex, socio-economic status, insurance status, smoking, obesity status, general health status, co-morbid cardiovascular conditions, and regional locations [13].

For the expenditures models we used a modified version of Aday and Andersen’s behavioral health model of health services use to estimate expenditures associated with CHC users compared to non-CHC users [15]. In other words, we used the variables specified in the Aday and Andersen behavioral health model as control variables in the expenditure models. The Aday and Andersen model hypothesizes that health expenditures or costs depend on predisposing, enabling, and health need factors. The predisposing factors were age, gender, race and marital status. The enabling factors were education, income and health insurance status. In our case, CHC is considered an enabling factor. The health needs factors were general health status and cardiovascular conditions such as high blood pressure, heart disease, angina, coronary heart attack and stroke. Smoking and obesity status were used as lifestyle factors. Census region and urban‒rural residence were used to measure location.

### Statistical Analysis

We estimated logistic regression models to determine the probability of receiving at least two HbA1c tests, a foot examination, or an eye examination within the past year, as indicators of quality of care. We converted the results into marginal effects from the mean for easier interpretation. Typically, a two-part model is utilized to estimate healthcare expenditures because some patients have no expenditures and a few of those who incur expenditures have very high expenses [16–21]. However, we used a one-part expenditure model to predict expenditures because nearly all patients with diabetes mellitus in the dataset had some type of expenditures for ambulatory care services. We used generalized linear models (GLM) with log link and gamma distribution to predict expenditures for CHC users compared to non-users of CHCs, conditional on patients with positive expenditures. Models were adjusted for the MEPS’ complex survey design and weighting by using the diabetes mellitus sample weight from the MEPS.

## Results

### Descriptive Statistics

On average, about 2 percent of patients with diabetes mellitus reported using a CHC. As shown in Table 1, overall an average of 81 percent of patients with diabetes mellitus reported receiving at least two HbA1c tests within the past year, 68 percent had a foot examination within the past year, and 59 percent had an eye examination within the past year. Chi square tests showed that there were no statistically significant differences among patients with diabetes mellitus who received at least two HbA1c tests or a foot examination within the past at CHCs compared to patients with diabetes mellitus who were non-users of CHCs. However, Chi square tests revealed that diabetes mellitus patients who were CHC users were less likely to report receiving an eye examination during the past year compared to diabetes mellitus patients who were non-users of CHCs (33% vs. 59%, p = 0.006). Patients with diabetes mellitus spent an average of $2,898 on ambulatory care. However, there were statistically significant differences in ambulatory care expenditures for patients with diabetes mellitus who were CHC users compared to patients with diabetes mellitus non-users of CHCs ($989 vs. $2,925, p = .0140), resulting in unadjusted savings of $1,936 per patient with diabetes mellitus receiving care in a CHC setting.

**Table 1:** Weighted Means/Proportions of Dependent and Independent Variables Used in the Models (N = 2,108), MEPS 2005–2008.

Variables	Total (N = 2,108)		CHC (N = 43)		Non-CHC (N = 2065)		P-value
	Mean/ Proportion	(S.E)	Mean/ Proportion	(S.E)	Mean/ Proportion	(S.E)	
**Dependent Variables**							
Received two or more HbA1c tests in past year	0.809	(0.011)	0.893	(0.075)	0.808	(0.011)	0.274
Received foot examination in past year	0.684	(0.139)	0.585	(0.132)	0.685	(0.014)	0.450
Received eye examination in past year	0.589	(0.137)	0.330	(0.094)	0.593	(0.014)	0.006
Amount of Ambulatory expenditures	$2898.18	(213.83)	$988.95	(721.32)	$2925.11	(216.68)	0.014
**Independent Variables**							
**Gender**							
Male	0.481		0.520		0.486		
Female	0.519	(0.019)	0.480	(0.130)	0.514	(0.018)	0.795
**Age (years)**							
Age	51.89	(0.324)	50.18	(1.977)	51.9	(0.326)	0.388
**Race/ethnicity**							
Non-Hispanic white	0.695	(0.016)	0.496	(0.124)	0.698	(0.016)	0.103
Black	0.133	(0.013)	0.115	(0.031)	0.133	(0.013)	0.564
Hispanic	0.142	(0.014)	0.389	(0.120)	0.139	(0.014)	0.041
Asian	0.029	(0.006)	0.000	(0.000)	0.029	(0.006)	0.001
**Education**							
No degree	0.165	(0.012)	0.342	(0.122)	0.163	(0.012)	0.139
High school degree	0.541	(0.017)	0.534	(0.121)	0.541	(0.018)	0.958
College degree	0.132	(0.012)	0.052	(0.065)	0.133	(0.013)	0.217
Graduate degree	0.070	(0.009)	0.000	(0.000)	0.071	(0.009)	0.001
Other degree	0.088	(0.010)	0.000	(0.000)	0.089	(0.010)	0.001
**Marital Status**							
Married	0.644	(0.018)	0.478	(0.128)	0.647	(0.018)	0.119
Divorced	0.151	(0.011)	0.212	(0.068)	0.150	(0.011)	0.366
Widowed	0.051	(0.006)	0.066	(0.067)	0.051	(0.006)	0.826
Separated	0.034	(0.005)	0.071	(0.080)	0.034	(0.005)	0.649
Never Married	0.119	(0.012)	0.173	(0.093)	0.118	(0.012)	0.557
**Income**							
≤ 100% of FPL	0.141	(0.011)	0.178	(0.052)	0.140	(0.011)	0.467
101–200% of FPL	0.175	(0.011)	0.591	(0.120)	0.169	(0.011)	0.001
201–400% of FPL	0.297	(0.011)	0.140	(0.069)	0.299	(0.017)	0.030
Over 400% of FPL	0.388	(0.017)	0.091	(0.086)	0.392	(0.017)	0.001
**Insurance Status**							
Private insurance	0.693	(0.016)	0.197	(0.084)	0.700	(0.016)	0.001
Public insurance	0.180	(0.123)	0.182	(0.086)	0.180	(0.012)	0.987
Uninsured	0.108	(0.010)	0.621	(0.109)	0.101	(0.009)	0.001
**Health Status/Condition**							
Fair/poor health	0.391	(0.015)	0.645	(0.102)	0.387	(0.015)	0.011
Obesity	0.635	(0.013)	0.706	(0.112)	0.634	(0.013)	0.529
Co-morbid cardiovascular conditions	0.703	(0.013)	0.853	(0.082)	0.701	(0.013)	0.073
Current smoker	0.196	(0.011)	0.095	(0.075)	0.197	(0.011)	0.180
**Region/Location**							
East	0.175	(0.018)	0.073	(0.048)	0.177	(0.018)	0.008
Midwest	0.227	(0.015)	0.172	(0.097)	0.228	(0.015)	0.556
South	0.376	(0.020)	0.368	(0.117)	0.376	(0.020)	0.946
West	0.221	(0.022)	0.387	(0.115)	0.219	(0.022)	0.142
Metropolitan Statistical Area (MSA)	0.813	(0.015)	0.895	(0.106)	0.812	(0.015)	0.432
**Year**							
2008	0.348	(0.010)	0.413	(0.066)	0.347	(0.010)	0.314
2007	0.184	(0.014)	0.166	(0.124)	0.184	(0.014)	0.886
2006	0.193	(0.012)	0.126	(0.080)	0.194	(0.012)	0.390
2005	0.275	(0.014)	0.295	(0.129)	0.275	(0.014)	0.879

The sample characteristics were fairly distributed among patients with diabetes mellitus who were CHC users compared to their counterparts who were non-users of CHCs. However, consistent with the literature, patients with diabetes mellitus who were CHC users compared to non-users of CHCs are less likely to have a graduate education (0% vs. 7%, p = 0.001), more likely to be poor, have a higher proportion of family incomes between 101–200% of the Federal Poverty Level (FPL) (59% vs. 17%, p = 0.01), have a lower proportion of family incomes greater than 400% of the FPL (9% vs. 39%, p = 0.001), are less likely to have private insurance (19% vs. 70%, p = 0.001), more likely to be uninsured (62% vs. 10%, p = 0.001), more likely to be in fair/poor health (64% vs. 39%, p = 0.001) and less like to live in the northeastern region of the United States (7% vs. 18%, p = 0.008).

### Multivariate Results

Marginal effects from multivariate logistic regressions showed that patients with diabetes mellitus who used CHCs were 12 percent more likely to receive at least two HbA1c tests in the past year compared to those patients with diabetes mellitus who used other settings of ambulatory care (Table 2). On the other hand, patients with diabetes mellitus who used CHCs were 3 percent less likely to receive a foot examination or an eye examination in the past 12 months compared to similar patients who used non-CHC settings. However, findings in variation of quality between CHC users and non-users of CHCs for all three indicators were not statistically significant for any of the indicators of quality of diabetes mellitus care used above. In terms of expenditures, the analysis considered only patients with diabetes mellitus with positive expenditures, which represented about 98% of the total sample. Results showed that patients with diabetes mellitus who used CHCs spent less on ambulatory care compared to similar patients who used other settings of ambulatory care (Table 2). Multivariate results that adjusted for socio-demographic variables, SES, health status, and health conditions measures showed that patients with diabetes mellitus who used CHCs saved payers and individuals about $1,656 in ambulatory care costs compared to non-users of CHCs.

**Table 2:** Results from Multivariate Models of Quality of Care and Expenditures for Patients with Diabetes Mellitus, MEPS 2005–2008.

Variables	Marginal Effects From Logistic Regressions (Quality Measures)			GLM
	HbA1c Test	Foot Examination	Eye Examination	Expenditures
	(S.E)	(S.E)	(S.E)	(S.E)
**CHC**	0.12	-0.03	-0.03	-0.87**
	(0.12)	(0.12)	(0.10)	(0.42)
**Gender** (Reference group is Male)				
Female	0.00	0.02	0.03	0.09
	(0.03)	(0.03)	(0.03)	(0.10)
**Age**				
Age	-0.01	-0.01	-0.02*	0.02
	(0.01)	(0.01)	(0.01)	(0.04)
Age-squared	0.00	0.00	0.00**	0.00
	(0.00)	(0.00)	(0.00)	(0.00)
**Race Ethnicity** (Reference group is White)				
African-American	0.03	0.03	-0.01	0.02
	(0.03)	(0.04)	(0.04)	(0.18)
Hispanic	-0.03	-0.08*	-0.06	-0.35**
	(0.04)	(0.04)	(0.05)	(0.15)
Asian	-0.05	-0.19	0.02	-0.37
	(0.10)	(0.12)	(0.10)	(0.37)
**Education** (Reference group is No Degree)				
High school degree	-0.02	0.00	0.04	-0.13
	(0.03)	(0.03)	(0.03)	(0.12)
College degree	-0.04	0.05	0.03	-0.01
	(0.05)	(0.05)	(0.05)	(0.20)
Graduate degree	-0.02	-0.02	0.06	0.00
	(0.08)	(0.08)	(0.10)	(0.26)
**Marital Status** (Reference group is Married)				
Divorced	-0.01	-0.02	-0.03	0.28*
	(0.05)	(0.04)	(0.05)	(0.15)
Widow	-0.04	0.00	0.02	-0.16
	(0.09)	(0.07)	(0.07)	(0.23)
Separated	0	-0.01	-0.07	0.34
	(0.12)	(0.08)	(0.07)	(0.46)
Never married	0.02	0.05	-0.04	0.17
	(0.05)	(0.05)	(0.05)	(0.22)
**Income Status** (Reference group is ≤ 100% FPL)				
100–199% FPL	-0.03	-0.05	0.01	-0.14
	(0.04)	(0.05)	(0.05)	(0.21)
200–400% FPL	0.01	0.00	0.06	-0.25
	(0.04)	(0.05)	(0.04)	(0.24)
Over 400% FPL	-0.01	0.06	0.09	-0.26
	(0.05)	(0.06)	(0.06)	(0.24)
**Insurance Status** (Reference group is Private Insurance)				
Public insurance	0.02	0.01	-0.01	-0.06
	(0.04)	(0.04)	(0.04)	(0.20)
Uninsured	-0.06	-0.08*	-0.19***	-0.67***
	(0.04)	(0.05)	(0.04)	(0.19)
**Health Status/Conditions**				
Fair/poor health	0.03	0.08**	-0.04	0.34***
	(0.02)	(0.03)	(0.03)	(0.12)
Obesity	-0.01	-0.04	0.01	0.00
	(0.03)	(0.03)	(0.03)	(0.12)
Current smoker	-0.01	-0.04	-0.07**	-0.11
	(0.04)	(0.04)	(0.03)	(0.13)
Co-morbidities	0.04	0.04	-0.03	0.23*
	(0.04)	(0.03)	(0.03)	(0.13)
**Region** (Reference group is Northeast)				
Midwest	0.01	0.04	-0.03	0.07
	(0.03)	(0.05)	(0.05)	(0.25)
South	0.02	-0.05	-0.08*	-0.15
	(0.03)	(0.04)	(0.04)	(0.23)
West	-0.01	-0.01	-0.03	-0.27
	(0.04)	(0.05)	(0.04)	(0.24)
MSA	-0.03	0.02	0.02	0.02
	(0.03)	(0.04)	(0.04)	(0.14)
**Year** (Reference group is 2005)				
2008	0.01	-0.07	0.01	-0.01
	(0.05)	(0.05)	(0.05)	(0.18)
2007	0.00	-0.06	0.02	0.00
	(0.04)	(0.03)	(0.04)	(0.15)
2006	0.01	-0.03	-0.02	0.04
	(0.03)	(0.04)	(0.04)	(0.15)
Constant				6.79***
				-0.97
**Goodness of Fit (GOF) Test**	Prob (Pearson)> χ^2^ = 0.5476	Prob (Pearson)> χ^2^ = 0.3805	Prob (Pearson)> χ^2^ = 0.3123	LRT Prob > (χ^2^) = 174.87
**N**	**2,186**	**2,680**	**2,716**	**3,083**

* p < .10

** p<0.05

*** p < .01

## Discussion

The objective of this study was to examine variations in the quality and cost of care provided to patients with diabetes mellitus by CHCs compared to other settings of primary care by using nationally representative data sets. Findings from this study advance the literature on quality of care and ambulatory costs for patients with diabetes mellitus in primary care settings by confirming that community health centers provide equivalent quality of care at lower costs to their patients. Results showed that while the quality of diabetes mellitus care between CHC users and non-users was similar, CHC users saved approximately $1,656 in ambulatory care compared to non-users of CHCs.

These findings are consistent with most studies in the literature that have found that the provision of care at CHCs is associated with significant savings while demonstrating that the quality of care provided by CHCs is comparable to other settings of primary care [2, 6–8]. Our results, however, are different from at least two studies in the literature. Chin et al. found that CHCs faced significant barriers to improving quality, access, and efficiency of care provided to patients with diabetes mellitus [22]. Additionally, a study by Beal and Hernandez found that CHC patients received lower quality of primary care compared to patients who received care in private offices [9]. The study by Beal and Hernandez used data from the 2006 Commonwealth Fund national survey of Health Care Quality to examine the importance of having a regular provider in CHCs for high quality care. The authors found that patients who reported receiving care from CHCs indicated lower rates of preventive services compared to those who reported receiving care from private doctors’ offices. However, the differences in quality of care between CHCs and private doctors’ offices were eliminated when CHC patients had a regular provider. Our study did not control for usual source of care in our quality of care models because, as an access variable, usual source of care may be correlated with quality of care through health insurance status [23]. Surprisingly, co-morbidities did not have much influence on the quality indicators. However, co-morbidities increased expenditures by 23 percentage points for patients with diabetes mellitus.

This study has a few limitations. Due to the cross-sectional design of the study, we are not able to infer causality. The analysis would have to consider and address in a rigorous manner important issues of sample selection, reverse causality and measurement error before drawing any causal inferences. For instance, the quality of care variables were also self-reported measures of process outcomes of diabetes mellitus care. Although we controlled for patients who reported poor/fair health or co-morbid cardiovascular conditions, it is possible that patients with severe diabetes mellitus conditions may be more likely to visit non-CHC providers more often and may be more likely to receive diabetes mellitus tests compared to those reporting excellent/good health and no co-morbid cardiovascular conditions. Use of CHCs was self-reported, and some patients may not be able to distinguish between CHCs and other types of health centers, potentially resulting in measurement bias of the CHC variable. This bias could have been reduced by identifying provider addresses in the MEPS to determine whether care was received at a CHC. However, the geocoded files in the MEPS that would allow completing such analysis are not publicly available.

We also acknowledge that the quality indicators were self-reported measures of process outcomes of diabetes mellitus care. We could not control for the severity level of diabetes mellitus because we had no information on glycemic control, achievement of HbA1c targets, or incidence of diabetes mellitus-related complications among patients. Although, recent data showed that there are about 23 million users of CHCs in the United States, it is important to note that only 2% of patients with diabetes mellitus used CHCs in our sample, which limits our ability to generalize our findings to the total population of patients with diabetes mellitus that use CHCs in the United States.

## Conclusion

This study shows that while there are no statistically significant differences in the quality of diabetes mellitus care received at CHCs compared to other primary care settings, CHC users saved approximately $1,656 in ambulatory care compared to non-users of CHCs. Assessing the mechanisms through which CHCs achieve cost savings while improving the quality of care is important because the current findings may suggest an opportunity for policymakers and clinicians to control costs without compromising the quality of care of diabetes mellitus in the U.S. For instance, it would be important to understand how CHCs save money. Are these savings due to better patient information and follow-up visits, shorter waiting times or better medical treatment? Another important and relevant question is how much the savings is out-of-pocket and how much of it should be allocated to different insurers such as private insurance and Medicaid. It would require the use of larger and longitudinal data sets to run separate models of expenditures for patients with health insurance and for out of pockets expenditures to answer these questions. Sample size limitations in the number of patients with diabetes mellitus that used CHCs precluded us from estimating separate models to answer these questions. Future research should aim to address these questions, particularly in the context of the Affordability Care Act of 2010 that seeks to aims to improve health outcomes for patients with diabetes while containing costs.

## Supporting Information

S1 Data Richard, Patrick (2015): Cost and Quality paper data. figshare. http://dx.doi.org/10.6084/m9.figshare.1533272.
(ZIP)
